# A novel live attenuated duck Tembusu virus vaccine targeting N7 methyltransferase protects ducklings against pathogenic strains

**DOI:** 10.1186/s13567-023-01170-0

**Published:** 2023-06-12

**Authors:** Xuedong Wu, Shanzhi Huang, Mingshu Wang, Shun Chen, Mafeng Liu, Dekang Zhu, Xinxin Zhao, Ying Wu, Qiao Yang, Shaqiu Zhang, Juan Huang, Xumin Ou, Ling Zhang, Yunya Liu, Yanling Yu, Qun Gao, Sai Mao, Di Sun, Bin Tian, Zhongqiong Yin, Bo Jing, Anchun Cheng, Renyong Jia

**Affiliations:** 1 Research Centre of Avian Disease, College of Veterinary Medicine of Sichuan Agricultural University, Chengdu, 611130 China; 2 Institute of Preventive Veterinary Medicine, Sichuan Agricultural University, Chengdu, 611130 China; 3 Key Laboratory of Animal Disease and Human Health of Sichuan Province, Chengdu, 611130 China

**Keywords:** DTMUV, live attenuated vaccine, N7-methyltransferase, immunogenicity, immunoprotection

## Abstract

Duck Tembusu virus (DTMUV), an emerging pathogenic flavivirus, causes markedly decreased egg production in laying duck and neurological dysfunction and death in ducklings. Vaccination is currently the most effective means for prevention and control of DTMUV. In previous study, we have found that methyltransferase (MTase) defective DTMUV is attenuated and induces a higher innate immunity. However, it is not clear whether MTase-deficient DTMUV can be used as a live attenuated vaccine (LAV). In this study, we investigated the immunogenicity and immunoprotection of N7-MTase defective recombinant DTMUV K61A, K182A and E218A in ducklings. These three mutants were highly attenuated in both virulence and proliferation in ducklings but still immunogenic. Furthermore, a single-dose immunization with K61A, K182A or E218A could induce robust T cell responses and humoral immune responses, which could protect ducks from the challenge of a lethal-dose of DTMUV-CQW1. Together, this study provides an ideal strategy to design LAVs for DTMUV by targeting N7-MTase without changing the antigen composition. This attenuated strategy targeting N7-MTase may apply to other flaviviruses.

## Introduction

Duck Tembusu virus (DTMUV), an emerging pathogenic flavivirus from the genus *flavivirus*, has become one of the main pathogens threatening duck industry in China, which can cause a significant decrease in egg production in laying ducks and neurological dysfunction and death in ducklings [1]. Flaviviruses are a group of enveloped positive-sense single-stranded RNA viruses, including Zika virus (ZIKV), dengue virus (DENV), Japanese encephalitis virus (JEV), tickborne encephalitis virus (TBEV), yellow fever virus (YFV), and West Nile virus (WNV). Most of flaviviruses are arboviruses transmitted by ticks or mosquitoes. They can infect different hosts, including humans, poultry, livestock, mice and other wild animals, causing a variety of diseases and posing a serious threat to people’s health, public safety and economic development. The Tembusu virus was first isolated from *Culex tritaeniorhynchus* in Kuala Lumpur, Malaysia in 1955 [2]. It was not until 2000 that a virus similar to Tembusu virus was isolated from chickens in Malaysia, known as Sitiawan virus (SV), which caused encephalitis and growth retardation in chickens [3]. In April 2010, there was an outbreak of severe duck egg-drop syndrome caused by DTMUV in China [4, 5], and the virus quickly spread to other avian species, such as pigeons, chickens [6], sparrows [7] and geese [8, 9], causing huge economic losses. DTMUV can be efficiently replicated in avian cell lines (e.g. DEF, CEF, GEF and DF-1), a variety of mammalian (e.g. BHK-21, HeLa, Vero and 293-T) and mosquito cell lines (e.g. C6/36) [2, 10]. Previous study has shown that serum antibodies against DTMUV were detected in duck farm workers and nucleic acid test of DTMUV was positive in oral pharyngeal swabs of some workers [11]. Recent study has reported that some people who are not exposed to ducks displayed high levels of anti-DTMUV IgG antibodies in their serum [12]. These studies suggest that DTMUV has the potential to infect people. Although no human infection with DTMUV has caused disease to date, the host spectrum of DTMUV is expanding, posing a serious threat to public health security and requiring highly effective vaccines or strict bio-security measures to control its spread.

DTMUV has an ~11-kb-length RNA genome with a 5′ type I cap structure [13]. The genome contains a single ORF that encodes a polyprotein and this polyprotein was processed by the NS2B-3 protease and cell host signalase to generate three structural proteins (capsid [C], premembrane [prM], and envelope [E]) and seven non-structural proteins (NS1, NS2A/NS2B, NS3, NS4A/NS4B, and NS5) [14, 15]. The N-terminus of NS5 protein contains a methyltransferase (MTase) domain that catalyzes the N7 and 2′-O methylations of viral RNA to form mature 5′-cap structure [16]. Notably, the mature 5′-cap structure is essential for RNA stability and efficient translation during flavivirus replication [17, 18]. Besides, flaviviral ribose 2′-O methylation assists viral RNA to evade recognition by host RNA sensors, such as RIG-I, thereby interfering with the host innate immune response [19, 20]. Flavivirus N7 methylation facilitates the translation of viral RNA to ensure viral proliferation [21]. K61-D146-K182-E218 catalytic tetrad is the conserved active site of flavivirus MTase. It has been shown that the flavivirus K61A, D146A, K182A, or E218A single mutation disables the 2’-O-MTase activity of the virus and significantly reduces the N7-MTase activity (the D146A mutation abolished N7-MTase activity) [21]. Our previous research has also shown that the DTMUV entire K61-D146-K182-E218 motif is essential for 2’-O-MTase activity and N-7-MTase activity requires only D146 [22]. In addition, flaviviruses with K61A, K182A, and E218A single point mutation showed attenuated virulence and significantly induced type I interferon (IFN) signaling pathway or were highly sensitive to type I IFN [19, 22]. DTMUV K61A, K182A, and E218A mutant viruses showed attenuated virulence and induced a higher innate immunity [17, 22]. YFV E218A mutant induced type I IFN production by improving the recognition ability of RIG-I for foreign antigens [19]. JEV E218A mutant was highly attenuated due to enhance sensitivity to type I IFN and IFITs [23]. DENV K61A and E217A mutants were highly sensitive to type I IFN inhibition [24]. Studies have shown that the weakened virulence of these MTase-deficient flaviviruses does not affect the immunogenicity of the virus, and generates strong humoral and cellular immunity in vivo, which can be used as an approach for vaccine design [23–25]. JEV E218A mutant was attenuated in mice and could protect animals from lethal challenge with virulent virus strains [23]. DENV K61A and E217A mutants were attenuated in mice and rhesus monkeys and elicited a strong adaptive immune response [24]. However, whether DTMUV K61A, K182A and E218A mutants are promising for the development of live attenuated vaccines (LAVs) remains unknown.

In this study, we investigated the immunogenicity and immunoprotection of N7-MTase-deficient DTMUV K61A, K182A and E218A in ducklings. We found that these three mutants were safe for duckling and significantly activated the host humoral and cellular immune responses, which ultimately protected duckling against the pathogenic strain. In a word, we provide a novel strategy to design LAVs for DTMUV by targeting N7-MTase without changing the antigen composition.

## Materials and methods

### Viruses, experimental animals, vaccine strains and antibodies

The wild-type (WT) DTMUV and K61A, K182A and E218A three mutant DTMUVs were rescued by our laboratory through the DTMUV reverse genetics system [22]. A clinical DTMUV CQW1 strain (KM233707) was isolated in 2013 by our laboratory [13]. The 5 day-old ducklings without specific pathogens were purchased from Shandong Haotai Experimental Animal Breeding Co., LTD (Jinan, China) and fed in isolators with negative pressure. The commercial live attenuated DTMUV vaccine WF100 strain was purchased from Shandong Qilu Animal Health Products Co., LTD (Jinan, China). The WF100 strain, produced by Shandong Qilu Animal Health Products Co., LTD, was obtained by serially passaging DTMUV WFG36 strain in chicken embryo fibroblasts cells (CEFs) to reduce its virulence. Mouse monoclonal antibody against E protein was prepared in our laboratory (unpublished). Fluorescein (FITC) Conjugate Goat Anti-Mouse IgG (H + L) was purchased from Thermo Fisher Scientific.

### Determination of virus titer

The virus titer was determined by fluorescence forming unit (FFU) method [22]. Briefly, BHK-21 cells cultured in 96-well plates were infected with 100 µL of virus fluids by DMEM diluted (10^− 1^-10^− 8^) (*n* = 4). After 72 h of infection, culture medium was discarded. The cells were fixed with 4% paraformaldehyde, permeabilized with 0.25% Triton X-100 PBS, and blocked with 5% BSA PBS, and then incubated with the mouse monoclonal antibody against E protein, followed by Fluorescein (FITC) Conjugate Goat Anti-Mouse IgG (H + L). The wells producing fluorescent spots were counted under fluorescence microscope and the fluorescence formative units were calculated according to the Reed-Muench methods.

### Virulence of mutants in vivo

To examine the virulence of mutants in vivo, 5 day-old specific pathogen free (SPF) ducklings were intramuscularly injected with 200 µL of DMEM containing 10^4.75^FFU/mL DTMUV WT virus or N7-MTase mutants or live attenuated DTMUV vaccine WF100 strain, while 200 µL of DMEM was intramuscularly inoculated as a mock control. Infected ducklings were monitored for clinical signs of disease, body weight change and mortality daily. At 2, 4, 6 days post-infection (dpi), sera were collected for detection of viral load. At 6 dpi, ducklings were sacrificed and brain and spleen were collected for histological analysis.

### Vaccination and challenge of ducklings

For immunization, 5 day-old SPF ducklings were intramuscularly vaccinated with a 200 µL volume containing either 10^4.75^FFU/mL DTMUV WT, K61A, K182A, E218A, WF100 vaccine strain or DMEM mock. At 3 weeks post-immunization (wpi), serum samples of infected ducklings were collected for detection of IFN-γ and IL-4 by enzyme-linked immunosorbent assay (ELISA). Serum anti-DTMUV IgG antibodies were measured by ELISA at 2, 3, 4, and 6 wpi. At 14 dpi, ducklings were challenged with 10^6^ FFU of DTMUV-CQW1 (a clinical isolated DTMUV strain) by intramuscular injection, then body weight change, clinical signs of disease and survival were monitored. At 4 days after the challenge, three ducklings in each group were euthanized. The spleen was then removed for histological analysis and serum were collected for detection of viral load.

### RNA extraction and RT-qPCR

The collected sera were used to extract viral RNA using TIANamp Virus DNA/RNA Kit (TIANGEN, Beijing, China), and then viral RNA was reversely transcribed into cDNA using PrimeScript™ RT reagent Kit with gDNA Eraser (TaKaRa, Dalian, China). Using the constructed quantitative standard curve of viral non-structural protein 3 (NS3), the virus copy numbers in sera were determined by qPCR according to TB Green^®^ Premix Ex Taq^™^ II (TaKaRa) instruction. The NS3 qPCR primers are as follows: DTMUV-NS3-qF: TAAAGAGGGAGCATACTGG, DTMUV-NS3-qR: GCAGGGTCTGTGAAGTGA. Reaction system of qPCR: TB Green: 5 µL, DTMUV-NS3-qF: 0.4 µL, DTMUV-NS3-qR: 0.4 µL, ddH_2_O: 3.2 µL, cDNA: 1 µL. qPCR reaction procedure: 95 ℃ 30s, 95 ℃ 5 s, 55.9 ℃ 30 s.

### Cytokine assay

The production of cytokines was measured by ELISA. Commercial duck IFN-γ and IL-4 sandwich ELISA kit (mlbio, Shanghai, China) was used to detect the production of Th1-type cytokine IFN-γ and Th2-type cytokine IL-4 in serum at 3 wpi following the manufacturer’s instruction.

### IgG titre measurement

An ELISA assay was conducted to measure the IgG antibody titre against DTMUV in the serum. Briefly, purified DTMUV was encapsulated in 96-well microtiter plates and then sealed with PBST containing 5% skim milk powder at 37 °C for 2 h, followed by five washes with PBST. The serum to be tested was diluted 1000 times with PBST containing 1% BSA and added into the plates for incubation at 37 °C for 1 h, followed by five washes with PBST. After that, HRP-conjugated goat anti-duck IgG antibodies (1:2000) (Sigma, St Louis, Mo, USA) were added and incubated at 37 °C for 1 h, followed by five washes with PBST. Finally, TMB single-component substrate solution (Solarbio, Beijing, China) was added to the plates and incubated for 15 min, then the reaction was terminated and the absorbance at 450 nm was determined by an ELISA plate reader (Bio-Rad, California, USA).

### Statistical analysis

The results were measured as a mean ± SD. The significance of the observed differences was assessed using one-way ANOVA and two-way ANOVA test with GraphPad Prism 8.0 software. Differences were considered significant when *P*-values < 0.05. The statistical significance of survival was analyzed using a survival curve and the log-rank (Mantel–Cox) test with GraphPad Prism 8.0 software, and significance was defined when *P*-values < 0.05.

## Results

### N7-MTase mutants are safe for ducklings

**Figure 1:**
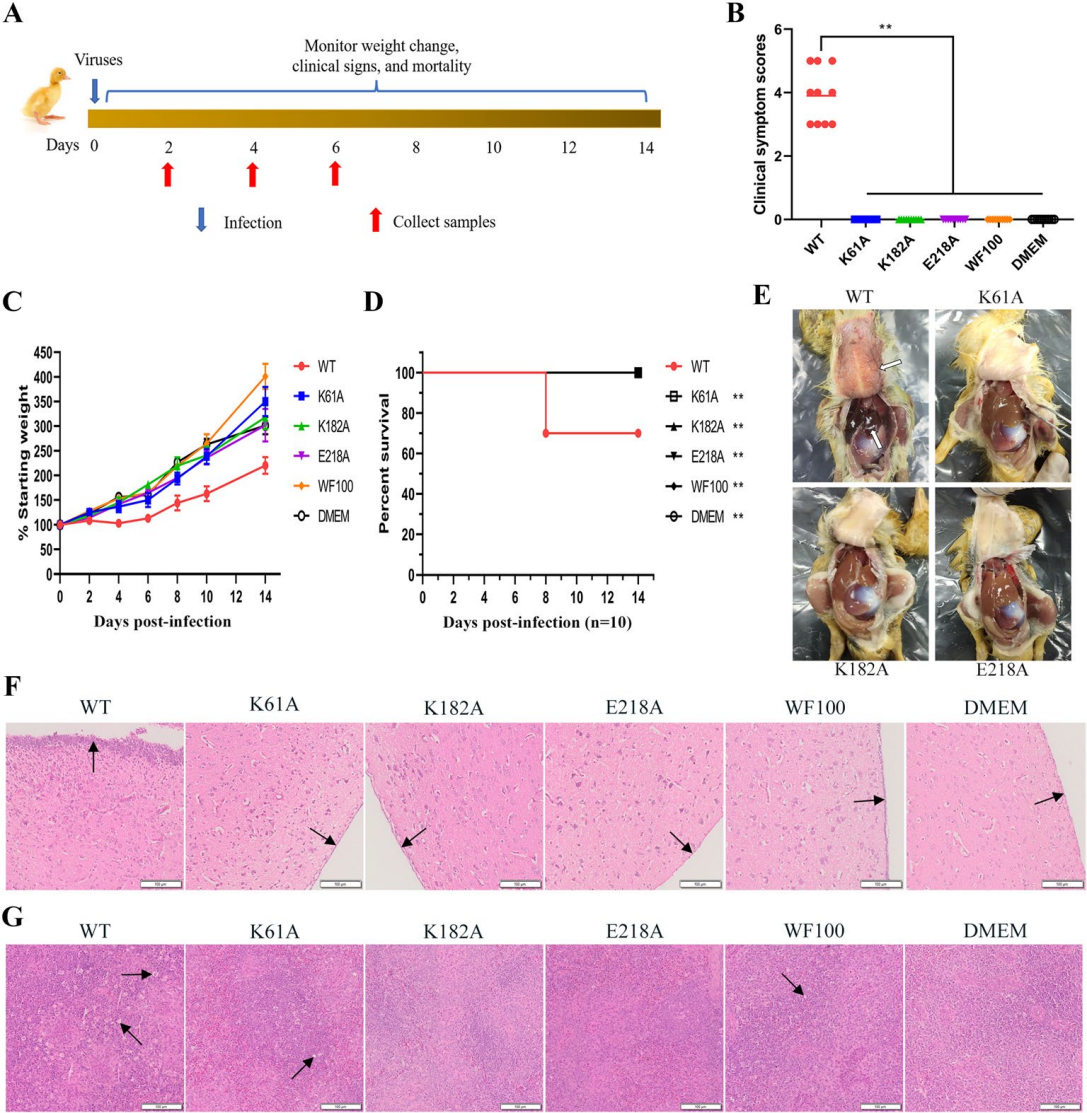
**
N7-MTase mutants are safe for ducklings**.** A** Scheme of infection. 5 day-old specific pathogen free (SPF) ducklings were intramuscularly injected with 200 µL of DMEM containing 10^4.75^FFU/mL WT virus or N7-MTase mutants or live attenuated DTMUV vaccine WF100 strain, while 200 µL of DMEM was intramuscularly inoculated as a mock control. Infected ducklings were monitored for 14 days for clinical signs of disease (*n* = 10), body weight changes (*n* = 3), and survival (*n* = 10). **B** clinical symptom scores. The data are presented as the means ± SDs. **p < 0.01 (one-way ANOVA). **C** body weight changes of ducklings. **D** Virulence of mutant viruses in ducklings. The statistical significance of survival was analyzed using a survival curve and the log-rank (Mantel–Cox) test, **p < 0.01. **E** Necropsy of ducklings of WT and mutant groups. **F**, **G** ducklings were sacrificed at 6 dpi, brain (**F**) and spleen (**G**) were collected for histological analysis. The black arrow indicates pathological injury. Scale bar, 100 μm.

To investigate the virulence and pathogenicity of the mutant viruses in vivo, groups of 5-day-old ducklings were intramuscularly inoculated with WT DTMUV, three mutant DTMUV strains (K61A, K182A and E218A) or live attenuated DTMUV vaccine WF100 strain, while DMEM was used as a mock control. Infected ducklings were monitored for clinical signs of disease, body weight change and mortality daily (Figure 1A). To better describe the clinical symptoms of infected ducklings, we established a clinical symptom score as follows: the score was 0 for no clinical symptoms, 1 for slowness of movement, 2 for instability of standing, 3 for hind limb palsy, 4 for paralysis and 5 for death. Clinical symptom scores are shown in Figure 1B. Hind limb palsy occurred in 4 ducks, paralysis occurred in 3 ducks, and death occurred in 3 ducks after WT DTMUV infection. No obvious clinical symptoms were observed in ducklings inoculated with three mutant DTMUV strains (K61A, K182A and E218A), live attenuated DTMUV vaccine WF100 strain and DMEM. Ducklings infected with K61A, K182A and E218A mutants showed a continuous increase in body weight, which was consistent with the increase trend of live attenuated DTMUV vaccine WF100 group and DMEM group and generally increased faster than that in WT virus group. While the body weight of the ducklings infected with WT virus did not increase in the first 6 days after infection, and began to increase slowly after 6 days (Figure 1C). As the infection progressed, the ducklings of K61A, K182A and E218A groups, WF100 vaccine group and DMEM group did not die within 0–14 days, and the survival rate was 100%. While three ducklings infected with WT virus died on day 8 after infection, with an eventual survival rate of 70% (Figure 1D). Necropsy of ducklings of WT group revealed hyperemia and hemorrhage in the blood vessels of the skin tissue and diffuse hemorrhage of liver, while ducklings infected with K61A, K182A and E218A viruses had no obvious lesions (Figure 1E). These results indicated that the virulence of K61A, K182A and E218A mutants to ducklings was sharply attenuated.

Further examination of brain and spleen injury also revealed significant attenuation of K61A, K182A and E218A mutants in ducklings. At 6 dpi, the brain and spleen of ducklings in each group were collected to make pathological sections, and histopathological changes were observed under the microscope. The observation of brain histopathological changes showed that no obvious abnormality was observed in ducklings inoculated with K61A, K182A and E218A mutant viruses (Figure 1F). The ducklings inoculated with live attenuated DTMUV vaccine WF100 strain showed slight meningeal thickening and meningeal inflammatory cell infiltration (Figure 1F). There were no pathological changes in brain in DMEM control group (Figure 1F). In contrast, the ducklings infected with WT virus showed typical symptoms of viral encephalitis, such as severe meningeal thickening and meningeal inflammatory cell infiltration, indicating that brain tissue had been seriously damaged (Figure 1F). By observing the pathological changes of spleen, it was found that the boundary between white pulp and red pulp was blurred in the spleen of ducklings infected with WT virus, and splenic lymphocytes were degenerated, necrotic, and vacuolated (Figure 1G). However, the boundary between red pulp and white pulp in spleen of K61A, K182A, E218A and WF100 vaccine groups was basically clear, and the spleen lymphocytes were slightly degenerated and necrotic (Figure 1G). There were no pathological changes in spleen in DMEM control group (Figure 1G). These results were correlated with clinical symptoms (Figure 1B) and weight changes (Figure 1C), further suggesting that the virulence of K61A, K182A and E218A mutants to ducklings was significantly reduced.

### N7-MTase mutants are replication-limited in ducklings

**Figure 2:**
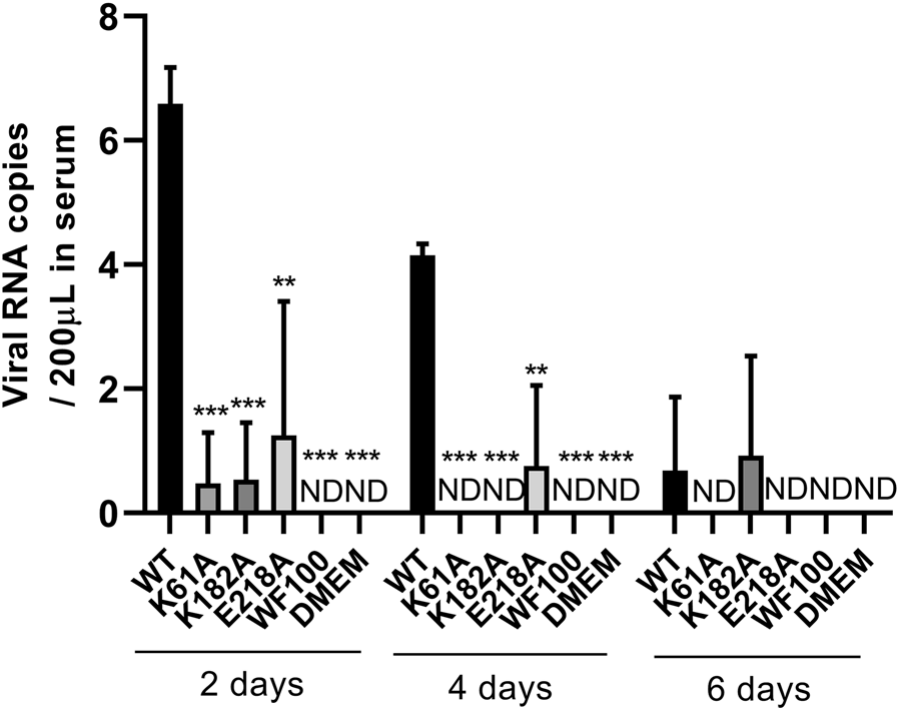
**
Viral copies number in sera at 2, 4 and 6 dpi were measured by RT-qPCR**. Each of these was conducted with three parallel replicates. Bars show means ± SDs. ***P < 0.001; **P < 0.01 (two-way ANOVA). ND, Not detected.

In order to explore the content changes of K61A, K182A and E218A mutants in ducklings, sera samples of ducklings in each group were collected at 2, 4 and 6 dpi to measure the copy numbers of the viruses by RT-qPCR. The results showed that ducklings infected with the WT virus presented significantly higher levels of viral copies number in the sera at 2, 4 and 6 dpi, whereas only a small level of viral copies number of ducklings infected with K61A, K182A and E218A mutant viruses could be detected at 2, 4 and 6 dpi and manifested as > 10^4^-fold decrease compared to WT virus-infected ducklings at 2 and 4 dpi (Figure 2). No virus was detected in WF100 vaccine group and DMEM group at 2, 4 and 6 days after inoculation (Figure 2). The virus copies number in the sera of WT, K61A and E218A groups reached the highest at 2 days after infection, and decreased gradually or even could not be detected as time went on. A small amount of virus load was detected in K182A group at 2 and 6 days after infection, and no virus was detected at 4 dpi (Figure 2). Together, these results indicated that the K61A, K182A and E218A mutants were significantly replication-limited in vivo and were more easily cleared by host animals.

### N7-MTase mutants induce cellular and humoral immune responses in ducklings

**Figure 3:**
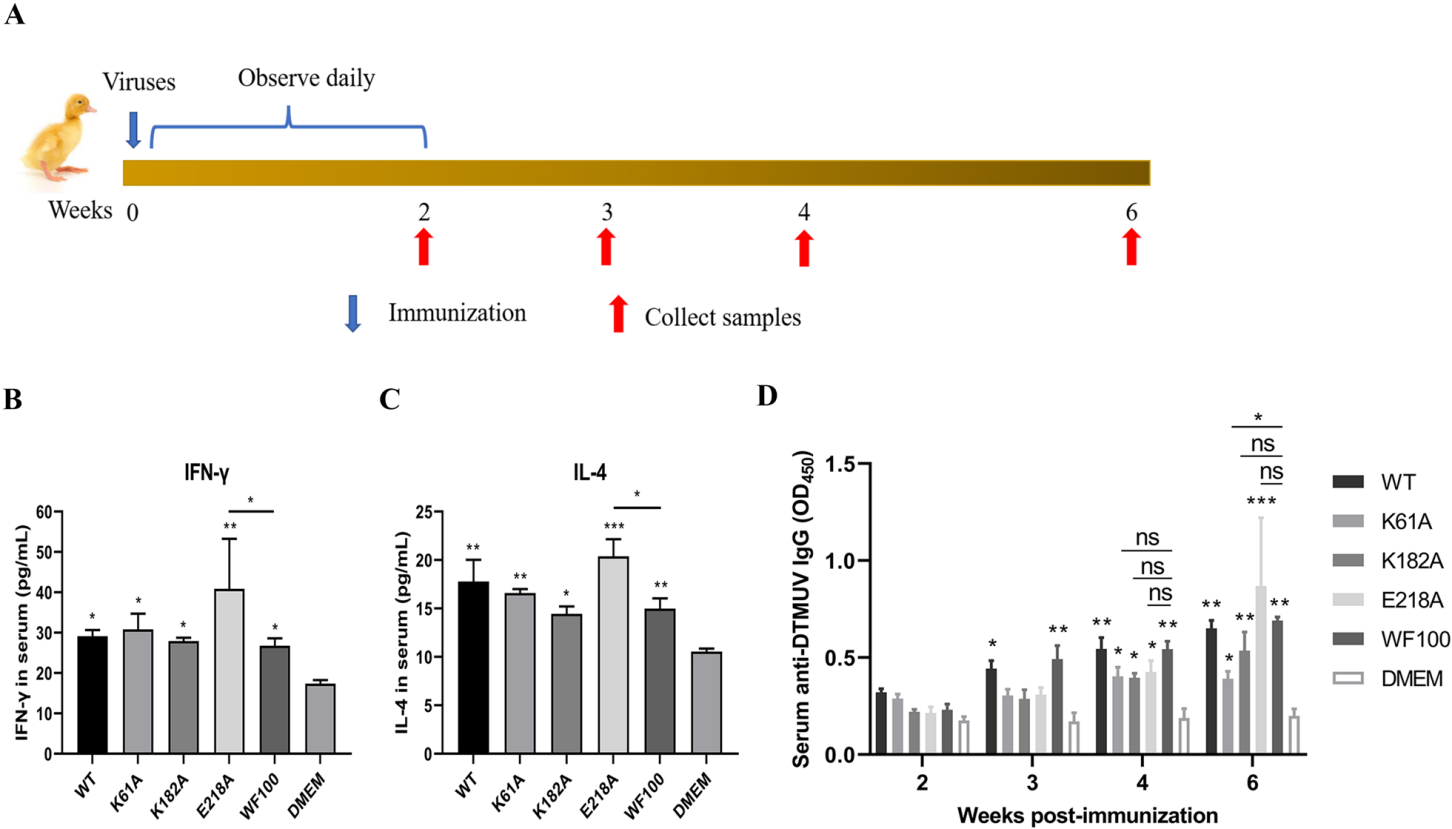
**
DTMUV N7-MTase mutants induce cellular and humoral immune responses in ducklings**.** A** Scheme of immunization. 5 day-old SPF ducklings were intramuscularly vaccinated with a 200 µL volume containing either 10^4.75^FFU/mL DTMUV WT, K61A, K182A, E218A, WF100 vaccine strain or DMEM mock. At 3 weeks post-immunization (wpi), serum samples of infected ducklings were collected for detection of IFN-γ and IL-4 by ELISA. **B** IFN-γ levels in serum. Each of these was conducted with three parallel replicates. Bars show means ± SDs. **P < 0.01; *P < 0.05 (one-way ANOVA). **C** IL-4 levels in serum. Each of these was conducted with three parallel replicates. Bars show means ± SDs. ***P < 0.001; **P < 0.01; *P < 0.05 (one-way ANOVA). **D** Serum anti-DTMUV IgG antibodies were measured by ELISA at 2, 3, 4, and 6 wpi. Each of these was conducted with three parallel replicates. Bars show means ± SDs. ***P < 0.001; **P < 0.01; *P < 0.05; ns, no significance, *P* > 0.05 (two-way ANOVA).

Based on the attenuation of N7-MTase mutants in ducklings, we further evaluated the immunogenicity and protective efficacy of the mutants in ducklings to investigate the possibility of targeting viral N7-MTase to design live attenuated vaccines. To investigate the effects of K61A K182A and E218A recombinant mutant DTMUV on the cellular and humoral immune responses in ducks, levels of Th1 cytokine IFN-γ, Th2 cytokine IL-4 and anti-DTMUV IgG antibody in the serum were determined by ELISA. As shown in Figure 3A, groups of 5-day-old SPF ducklings were intramuscularly inoculated with a 200 µL volume containing either 10^4.75^FFU/mL DTMUV WT, K61A, K182A, E218A, WF100 vaccine strain or DMEM mock. At 3 wpi, serum samples of immunized ducklings were collected for evaluation of cellular immune responses. The results showed that the levels of IFN-γ and IL-4 in the serum of ducklings infected with WT, K61A, K182A, E218A or WF100 virus were significantly higher than those in DMEM group (Figures 3B and C). Unexpectedly, the levels of IFN-γ and IL-4 in the serum of ducklings in E218A group were significantly higher than those in WF100 vaccine group (Figures 3B and C). These results indicated that DTMUV N7-MTase mutants K61A, K182A and E218A could induce robust T cell response in ducklings, and the E218A mutant virus had the best induction effect. In addition, we determined the level of anti-DTMUV IgG antibody in serum at 2, 3, 4, and 6 wpi. The results showed that DTMUV-specific IgG induced by N7-MTase mutants K61A, K182A, and E218A rapidly reached a comparative level to the WF100 vaccine at 4 wpi and this could be maintained until 6 wpi (Figure 3D). Taken together, our findings suggest that DTMUV N7-MTase mutants K61A, K182A, and E218A can induce robust cellular and humoral immune responses in ducklings.

### Immunization with N7-MTase mutants protects SPF ducks from death

**Figure 4:**
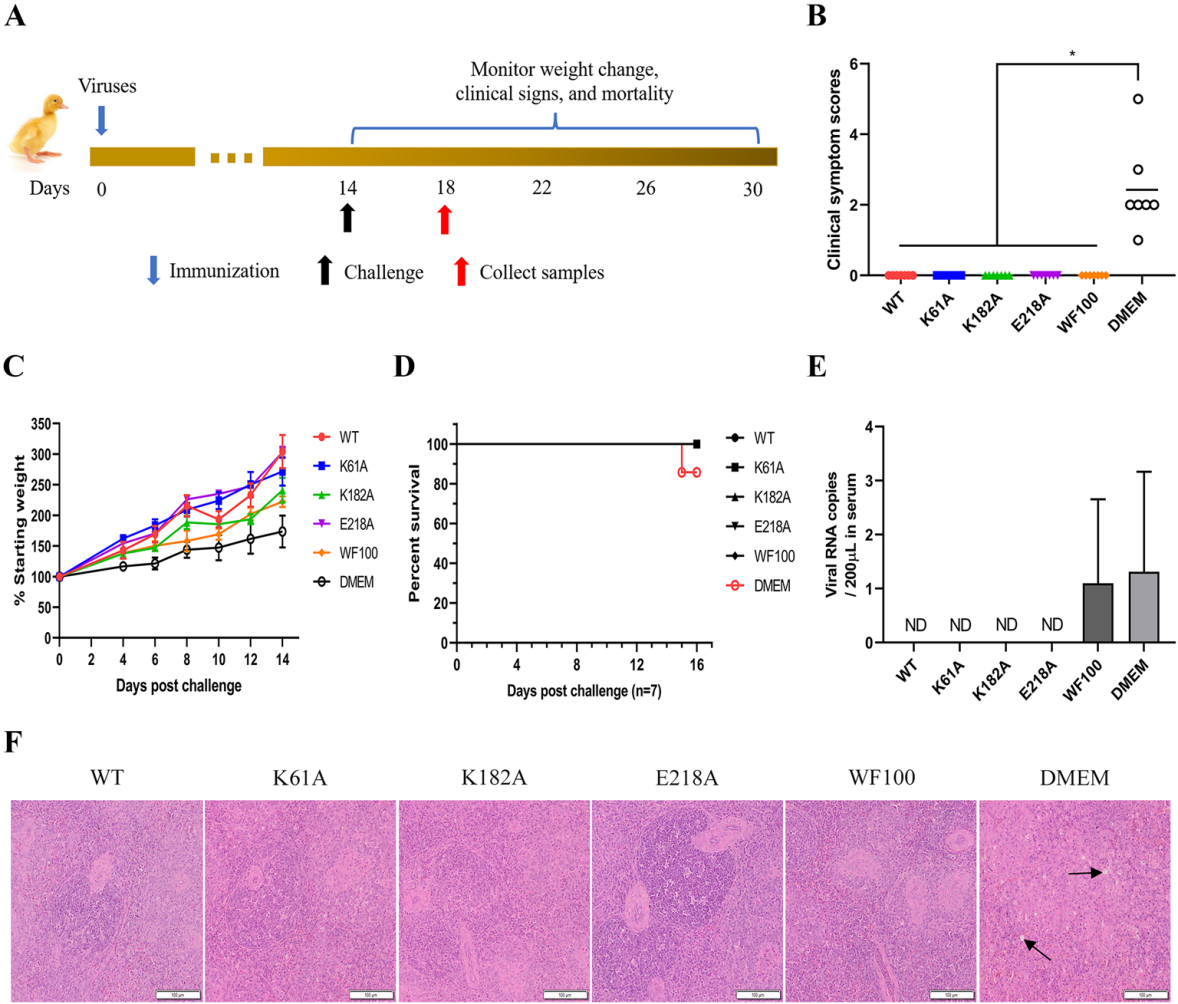
**
DTMUV N7-MTase mutants immunization protects ducks from death after challenge**.** A** Scheme of challenge of SPF duck. 5-day-old SPF ducklings were immunized with 200 µL 10^4.75^ FFU/mL WT, K61A, K182A, E218A, while the WF100 vaccine and DMEM were used as positive and mock control, respectively. At 14 dpi, animals were challenged with a high lethal dose of 10^6^ FFU WT DTMUV, then clinical signs of disease, body weight change and survival were monitored. **B** Clinical symptom scores (*n* = 7). The data are presented as the means ± SDs. *p < 0.05 (one-way ANOVA). **C** Body weight change (*n* = 3). Means with SD are shown. **D** Survival 16 days after challenge (*n* = 7). The statistical significance of survival was analyzed using a survival curve and the log-rank (Mantel–Cox) test. **E** Viral loads in sera were measured by RT-qPCR at 4 days post-challenge. Each of these was conducted with three parallel replicates. Bars show means ± SDs. ND, Not detected (one-way ANOVA). **F** Spleen histological changes of ducks. The black arrow indicates splenic lymphocytes were degenerated, necrotic, and vacuolated. Scale bar, 100 μm.

To determine the protective efficacy of N7-MTase mutants, we immunized ducklings as mentioned above, and then they were challenged with DTMUV-CQW1 (a clinically isolated DTMUV strain) at a lethal dose of 10^6^ FFU by the intramuscular route at 14 dpi (Figure 4A). The clinical symptoms of the challenged ducklings were evaluated according to the clinical scoring criteria mentioned above. As shown in Figure 4B, after challenge, 1 duck in DMEM group showed slow movement, 4 ducks showed unstable standing, 1 duck showed hind limb palsy and 1 duck died. whereas ducks in WT group, K61A group, K182A group, E218A group and WF100 group had no obvious clinical symptoms. Furthermore, mock-immunized ducks showed slow weight gain and one of the ducks died at 15 days after the challenge, whereas all the virus-immunized ducks showed rapid weight gain and survived the lethal challenge (Figures 4C and D). Moreover, viral load detection showed that DTMUV was not detected in the sera of WT-, K61A-, K182A- or E218A-immunized ducks at 4 days after the challenge, whereas a total of about 10^1.5^ copies of the virus was maintained in the sera of WF100- or DMEM-immunized ducks (Figure 4E). Similar results were shown in the spleen histopathological analysis (Figure 4F), in which no spleen damage was observed in the WT-, K61A-, K182A-, E218A- or WF100-immunized group after challenge with high pathogenic DTMUV-CQW1. Thus, these results indicated that single immunization with N7-MTase mutants could completely protect ducks from lethal challenges.

## Discussion

An increasing number of flaviviruses have been discovered from different contagious diseases, which have posed a significant threat to global public health, and a recent example is the outbreak of ZIKV throughout the Americas [26]. Although important advances have been made on the origin of flaviviruses, pathogenesis, replication, cross-species transmission and virus–host interactions, there is still lack of effective specific medical treatment against flaviviruses infection. Hence, safe and efficacious vaccination remains the most promising method for preventing viral infection and spread.

Nowadays, available strategies to develop flaviviruses vaccines include inactivated vaccines, subunit protein vaccines, LAVs, recombinant viral vector vaccines, RNA vaccines, and DNA vaccines, among others [27, 28]. LAVs typically consists of weakened versions of pathogens that can replicate defectively but closely mimic the protective immunity induced by live pathogens in the host, so that even a single immunization can elicit strong and long-lasting adaptive immune responses [29]. Successful experience in flaviviruses vaccine development started with YFV and JEV prevention in the last century. Live attenuated YFV vaccine was obtained by serially passaging pathogenic strains in chicken embryo to remove its neurotropic properties [30]. A live attenuated JEV vaccine has been available in China since 1988, and single immunization exhibited ≥ 95% efficacy [31, 32]. Unfortunately, traditional LAVs production is time-consuming and laborious, making it difficult to respond to public health emergencies. In addition, since their attenuation mechanism is not clear and they may recombine with other circulating flaviviruses, there is a risk of virulence recovery. While other types of vaccines are relatively safe, they have many drawbacks, such as poor immunogenicity, inability to effectively activate T-cell responses, the need for multiple immunizations, and high cost. Considering cost, timeliness, and effectiveness, a more operationally convenient, secure, affordable, and fully protected LAV would be optimal. The use of reverse genetic systems to rapidly introduce specific mutations into the viral genome to weaken the virus is a feasible and efficient way to develop live attenuated flavivirus vaccines, which can quickly respond to public health emergencies [33].

In the present study, the core functional domain of DTMUV MTase was targeted and three conserved key activity residues at positions K61, K182 and E218 of DTMUV MTase were mutated to rationally design live attenuated flavivirus vaccine by the reverse genetic system. Our results showed that the MTase mutants DTMUV K61A, K182A, and E218A were highly attenuated in both virulence and proliferation of the virus in ducklings, indicating that these mutants are very safe. More importantly, a single-dose immunization with K61A, K182A or E218A could induce humoral immune responses and produce robust T cell immune responses, which play an important role in virus clearance by the host. The protective effect of the MTase-deficient DTMUV K61A, K182A and E218A was similar to that of the commercial live attenuated DTMUV vaccine WF100, indicating that the N7-MTase mutants have the potential to develop into live attenuated DTMUV vaccines. Advantageously, the ducks in the WF100 group may have failed to clear out the wild-type virus in time, while the ducks in the other viruses immunized group cleared the wild-type virus faster than those in the WF100 group. In other flaviviruses, JEV E218A mutant was attenuated in mice and could protect animal from lethal challenge with virulent virus strains [23]. DENV K61A and E217A mutants were attenuated in mice and rhesus monkeys and elicited a strong adaptive immune response [24]. These studies further suggest that N7-MTase-deficient flaviviruses have the potential to develop into live attenuated vaccines.

In the cases of natural DTMUV infection, younger ducks and geese were more susceptible to the virus and showed more serious symptoms, lesions and higher mortality than the older flocks. The significant effect of age on the pathogenicity of DTMUV in ducks was confirmed by a series of experimental data. Studies have shown that DTMUV can cause severe clinical symptoms, neurological dysfunction, and even death in one-week-old ducks, with a mortality rate of about 30% [34]. Whereas DTMUV only causes mild clinical symptoms and no death in ducks older than 3 weeks of age [34]. Similar results were observed in DTMUV-infected 5- and 20-day-old goslings [9]. Age can affect the pathogenicity of DTMUV in animal, and younger animals are more susceptible to the virus. This precisely explains why the clinical strain CQW1 failed to cause a large number of ducks to die after challenge.

In conclusion, we have demonstrated that the highly debilitated N7-MTase mutant has potential as a live attenuated DTMUV vaccine candidate strain. This study provides a novel idea for the development of flavivirus vaccine. More importantly, we could further enhance the safety of the N7-MTase mutant by combining other mutations in viral proteins, such as T367K or M304R mutations in E protein for DTMUV [35, 36], to improve the probability of successful development of LAVs.

## Data Availability

The datasets during and/or analysed during the current study available from the corresponding author on reasonable request.
